# The nine stages of skin‐to‐skin: practical guidelines and insights from four countries

**DOI:** 10.1111/mcn.13042

**Published:** 2020-06-16

**Authors:** Kajsa Brimdyr, Karin Cadwell, Kristin Svensson, Yuki Takahashi, Eva Nissen, Ann‐Marie Widström

**Affiliations:** ^1^ Maternal Child Health I Center for Breastfeeding Healthy Children Project, Inc. USA; ^2^ Antenatal and Maternity Department Karolinska University Hospital Sweden; ^3^ Health Science Department Nagoya University Graduate School of Medicine Japan; ^4^ Department of Women's and Children's Health Karolinska Institute Sweden

**Keywords:** birth, breastfeeding, breastfeeding initiation, evidence‐based practice, interaction, newborn feeding Behaviours

## Abstract

Incorporating systematic evidence with clinical expertise is a key element in the quest to improve quality of care and patient outcomes. The evidence supporting skin‐to‐skin contact in the first hour after birth is robust and includes significantly improved outcomes for both mother and infant. This paper compares available iterative data about newborn behaviour in the first hour after birth to further describe the observable behaviour pattern and to provide clinical insight for further research. Although the evidence for positive outcomes through skin‐to‐skin contact are robust, there is a dearth of research specifically focused on clinical practice. The methodology considers the four available data sets that used Widström's 9 stages, which consists of studies from Japan, Sweden, Italy and the United States, examining the parameters of each stage across settings from around the world. This research provides an expanded understanding of the timing of the newborn's progression through Widström's 9 observable stages. We found that newborns in all four data sets began with a birth cry and continued through the remaining stages of relaxation, awakening, activity, rest, crawling, familiarization, suckling and sleeping during the first hours after birth and consolidated the data into a Sign of the Stages chart to assist in further research. The evidence supports making a safe space and time for this important newborn behaviour. Clinical practices should encourage and protect this sensitive period.

**Key messages**

- This paper considers the data about newborn behaviour in the first hour after birth from four countries, Sweden, Italy, Japan and the United States, to further describe the distinctive and observable behaviour pattern in order to assist clinicians and researchers to recognize the expectations and parameters of each of the nine stages.
- The behaviour of the newborn infant while skin‐to‐skin during the first hour or so after birth varies little from one practice setting to another.
- Clinical practices should encourage and protect this unique and important time by ensuring adherence to Step 4 of the BFHI, including immediate, continuous and uninterrupted skin‐to‐skin contact between all mothers and newborn infants during the first hour after birth.

## 1 INTRODUCTION

The evidence supporting the practice of skin‐to‐skin contact (SSC) after birth is robust indicating multiple benefits for both mother and baby; the 2016 Cochrane Review supports using immediate or early SSC to promote breastfeeding. (Moore, Bergman, Anderson, & Medley, 2016) Advantages for the mother include earlier expulsion of the placenta (Marín Gabriel et al., 2010), reduced post‐partum bleeding (Dordević, Jovanović, & Dordević, 2008) and lowered maternal stress levels (Handlin et al., 2009). The mother's oxytocin rises over the course of the first hour after birth (Nissen, Lilja, Widström, & Uvnäs‐Moberg, 1995) which in turn leads to more social interactive behaviours, including maternal caring behaviours (Kerstin Uvnäs‐Moberg et al., 2019) and may also promote parenting behaviours (K Uvnäs‐Moberg, 1989) (Winberg, 2005), bonding and attachment (Affonso, Wahlberg, & Persson, 1989). Advantages for the baby include a decrease of the negative consequences of the stress of being born (Bystrova et al., 2003; Lagercrantz & Slotkin, 1986; Takahashi & Tamakoshi, 2018; Takahashi, Tamakoshi, Matsushima, & Kawabe, 2011), more optimal thermoregulation, (Beiranvand, Valizadeh, Hosseinabadi, & Pournia, 2014; Bystrova, 2008), continuing even in the first days (Bystrova, 2008; Nimbalkar et al., 2014) and less crying (Christensson et al., 1992). SSC has been shown to increase breastfeeding initiation and exclusive breastfeeding while reducing formula supplementation in hospital, leading to an earlier successful first breastfeed (Bramson et al., 2010; Crenshaw et al., 2012; Mahmood, Jamal, & Khan, 2011; Marín Gabriel et al., 2010; Mikiel‐Kostyra, Mazur, & Bołtruszko, 2002; Srivastava, Gupta, Bhatnagar, & Dutta, 2014), as well as more optimal suckling (Righard & Alade, 1992).

Despite the research and compelling directives from world authorities, the implementation of immediate, continuous and uninterrupted SSC for all healthy mothers and newborns, regardless of feeding choice, has not become standard practice. According to UNICEF, only 45% of newborns experienced early SSC and breastfeeding (UNICEF, 2016). In the United States, 83% of hospitals reported that SSC was being practiced, according to the most recent CDC National Survey of Maternity Practices in Infant Nutrition and Care (Table 1.1a) (Centers for Disease Control and Prevention, n.d.). However, the question asked is ‘How many patients experience mother–infant skin‐to‐skin contact for at least 30 min within 1 h of uncomplicated vaginal birth?’ or ‘How many patients experience mother–infant skin‐to‐skin contact for at least 30 min within 2 h of uncomplicated caesarean birth?’ This does not meet the definition of immediate, continuous and uninterrupted SSC as described by WHO or UNICEF. Indeed, the most recent Cochrane review of early SSC for mothers and their healthy newborn infants highlights the inconsistencies of practice throughout the published research, reflecting the inconsistencies in clinical practice throughout the United States and the world. Only 47% of the eligible trials reported ‘early’ or ‘immediate’ SSC. The duration was inconsistent as well, from 15 min to 30 h (Moore et al., 2016).

Skin‐to‐skin care, on the other hand, implies a process, consistent with immediate, uninterupted contact between the mother and baby for an hour or more after the birth, during which maternal and infant assessments are incorporated, and the newborn goes through nine observable stages. These stages were first described by Widström (A. M. Widström et al., 1987) and later illustrated with a video (Widström, Ransjö‐Arvidsson, & Christiansson, 2010), two expanded papers (Widström et al., 2011; Widström, Brimdyr, Svensson, Cadwell, & Nissen, 2019) and a video directed at staff implementation (Brimdyr, Widström, & Svensson, 2011): Birth Cry, Relaxation, Awakening, Activity, Crawling, Resting, Familiarization, Suckling and Sleeping (Figure 1) during the first hour after birth (Brimdyr et al., 2011; Widström et al., 2010; Widström et al., 2011; Widström et al., 2019). The nine stages recognize the work of the mother and baby during this time, the complex system of hormones, instinctive behaviours and bonding, which goes beyond the implied passivity of simply putting the newborn skin‐to‐skin for a short time. There is work, and competent behaviours, that occur during this vital and unique time. Skin‐to‐skin care supports a newborn's exploration and self‐attachment, a result of the newborn's instinctive survival behaviour to find the breast and start suckling within an hour or so of the birth, leading to increased breastfeeding at discharge (Bramson et al., 2010), increased breastfeeding self‐efficacy (Aghdas, Talat, & Sepideh, 2014) and more optimal latch (Righard & Alade, 1992) and bypasses the issues associated with ‘hands‐on’ or forced latch (Svensson, Velandia, Matthiesen, Welles‐Nyström, & Widström, 2013).

Although the research and evidence are robust, there is a dearth of iterative research specifically focused on clinical practice. Recently, implementation of clinical practice was clarified to address the lag between research knowledge and clinical practice, to translate existing knowledge and guide clinician behaviour change (Widström et al., 2019). This paper contributes to the field by examining the worldwide similarities of newborn behaviour when skin‐to‐skin immediately after birth and defines the expectations and parameters of each of Widström's 9 stages in order to provide a practical guideline for clinicians and researchers.

## 2 METHODS

In this manuscript, previously published data on the observable behaviours of the newborn during the first hour after birth when placed skin‐to‐skin with mother immediately after birth were compared between different groups of infants, and median and quartiles were used, if available. Some videos from the United States and Japan were reanalysed by research assistants.

A literature review found only four studies (Brimdyr, Cadwell, Stevens, & Takahashi, 2017; Brimdyr et al., 2015; Dani et al., 2015; A‐M Widström et al., 2011) from Japan, the United States, Italy and Sweden, respectively, which have reported on the nine stages of newborn behaviour in relation to healthy newborn babies, born vaginally. Each hospital followed the World Health Organization guidelines for SSC between mother and newborn. Ethical permission for each study is reported in the original papers.

Widström et al. report on a convenience sample of 28 clinically uncomplicated vaginal birth Swedish newborns, many of whom were exposed to meperidine, epidural, pudendal block and so forth in labour (Widström et al., 2011). The median and quartiles are published and available (Widström et al., 2011). Dani et al. report on 17 clinically uncomplicated vaginal birth Italian newborns without any labour analgesia exposure (Dani et al., 2015) with a prospective observational controlled study and published the median and quartiles. We have previously reported on a convenience sample of 13 clinically uncomplicated vaginal birth Japanese newborns with no labour analgesia exposure (Brimdyr et al., 2017) but did not publish the median or quartiles. For the purposes of this paper, we are reporting on the 11 clinically uncomplicated vaginal birth American newborns with no labour analgesia exposure, a subgroup of the convenience sample of 63 dyads in an observational study reported on elsewhere (Brimdyr et al., 2015; Cadwell, Brimdyr, & Phillips, 2018). Previous papers did not report on the median or quartiles of the American newborns.

The data used in this paper from the Swedish and Italian studies have been published elsewhere (Dani et al., 2015; Widström et al., 2011). New data are presented from the United States and Japanese studies (Brimdyr et al., 2017, 2015). Existing videos were reanalysed in relation to the newly developed Sign of the Stage chart (Table 1). Education of the research assistants was conducted using a professional video (Brimdyr et al., 2011) and a workshop about Widström's 9 stages of newborn behaviour to ensure recognition of each stage. MAXQDA 11.0.2, 2013, a professional qualitative data analysis software, was used to separately and independently code the video recordings for Widström's 9 stages. Any inconsistencies required review and consensus. The Japanese videos and American videos were recoded to focus on the initial entry into each stage. The data were then charted onto the Sign of the Stage chart (Table 1), which could be used to generate the median and quartile data (Table 2).

**TABLE 1:** Nine stages for clinical practice: A chart to aid in the understanding of when a stage begins during skin‐to‐skin contact with mother and summary of maximum and minimum quartiles from four studies

Widström's stage	Timing	Sign to indicate entry into stage (must do at least one)
Birth Cry	(0–0)	Birth cry/sound as the lungs expand
Relaxation	(1–6)	Stillness
Awakening	(1–14)	Small thrusts of head (side to side or up and down)
		Small movements of limbs and shoulders Mouth activity
Activity	(4–25)	Moves limbs
		Moves head
		Determined movements
		Pushes without moving/rocking
		Roots
		Hand breast mouth movement
		Active hand movements around mouth
Crawling	(18–54)	Pushes/slides/leaps/moves which results in shifting body to the breast and nipple
Resting (first time)	(13–46)	Pauses within a stage or between stages
Familiarizing	(24–62)	*At the mother's breast/nipple area*
		Makes soliciting sounds
		Moves hand to mouth to breast
		Puts thumb/finger in mouth
		Touches tongue to nipple
		Massages breast
		Roots on breast
		Mouths breast
		Licks nipple
		Tongues hand
		Repeatedly licks nipple/suck attempts
Suckling	(39–90.3)	Self‐attaches and suckles
Sleep	(52.5–108)	Falls asleep

**TABLE 2:** Number of babies reported in each behavioural phase during skin‐to‐skin contact in the United States, Japan, Italy and Sweden studies

	United States (*n* = 11)	Japan (*n* = 13)	Italy (*n* = 17)	Sweden (*n* = 28)
Birth Cry	11	13	17	28
Relaxation	11	13	13	24
Awakening	11	13	17 (head movement)	28 (head movement)
Activity	10	13	17	28
Resting	8	5	17	25
Crawling	9	7	16	21
Familiarization	6	4	11	18
Suckling	5	2	7	15
Sleeping	1	0	10	28

For the Japanese and United States data, Excel (Microsoft Standard 2019, version 1808) was used to determine the count, median and quartiles of the data. SPSS Analysis of the video recordings provided data on the frequency of each stage and the time of appearance of in each of Widström's 9 stages. The data regarding the Swedish and Italian newborns were obtained from tables and text published in peer reviewed journals.

### 2.1 Ethical considerations

The ethical approval for all four studies were published separately (Brimdyr et al., 2017, 2015; Dani et al., 2015; Widström et al., 2011).

## 3 RESULTS

The number of babies reported in each behaviour phase in the studies from the United States, Japan, Italy and Sweden are documented in Table 2. All of the newborns experienced a Birth Cry. All of the newborns in the United States and Japan experienced Relaxation, with 13/17 of the Italian newborns and 24/28 of the Swedish newborns experiencing that stage. The Awakening Stage was reported differently in the Swedish and Italian studies—as elements of the stage rather than the stage itself. As a result, we have chosen to report on an element of Awakening, Head Movements, in the chart, illustrating that all of newborns in all four studies experienced the Awakening Stage. While only 10/11 of newborns in the United States study experienced the Activity Stage, all of the newborns in Japan, Italy and Sweden experienced that Stage. Only 5 of the 13 Japanese newborns rested during the first hour, while 8 of the 11 United States newborns, all of the Italian newborns and 25 of the 28 Swedish newborns experienced the Resting Stage. Nine of the 11 United States newborns, 7 of the 13 Japanese newborns, 16 of the 17 Italian newborns and 21 of the 28 Swedish newborns experienced the Crawling Stage. Within the first hour after birth, 5 of the 11 American newborns suckled, while only 2 of the 11 Japanese newborns self‐attached. Seven of the 17 Italian newborns self‐attached and suckled, and 15 of the 28 Swedish newborns met this. Only one American newborn was documented sleeping, and no Japanese newborns slept in the first hour after birth. Ten of the Italian newborns fell asleep and all of the Swedish newborns fell asleep. The median time of appearance (in minutes) of each stage, including the interquartile range (25th–75th quartile), is in Table 3, which reflects the data reported by each study.

**TABLE 3:** Median time at appearance (minutes) from birth of behavioural stage during skin‐to‐skin contact and interquartile range (25th–75th quartile)

Stage/country	United States (*n* = 11)	Japan (*n* = 18)	Italy (*n* = 17)	Sweden *(*n* = 28)
Birth Cry	0 (0–0)	0 (0–0)	0 (0–0)	0 (0–0)
Relaxation	3 (2–5)	2 (1–3)	3 (1–6)	2 (2–4)
Awakening	10 (6–14)	6 (4–8)		
Activity	22 (12–24)	11 (9–17)	13 (10–25)	8 (4–12)
Rest First Time	43 (28–46)	37 (36–41)	20 (15–38)	18 (13–26.50
Crawling	33 (33–36)	30 (26–35)	28.5 (21–48)	36 (18–54)
Familiarization	42 (33–48)	42 (41–44)	46 (24–52)	43 (29–62)
Suckling	52 (50–56)	52 (49–54)	45 (39–56)	62 (43.5–90.3)
Sleeping	59	N/A	94 (78–108)	70 (52.5–79)

## 4 DISCUSSION

All of the newborns reported on in the United States, Japan, Italy and Sweden experienced some version of a Birth Cry. Despite its name, the Birth Cry Stage is about more than just noise. It is the time when the newborn's lungs expand, transitioning from in‐utero reliance on the placenta for oxygen to a time of breathing air. Although this is traditionally noticed through a noisy cry, the transition to breathing air can sometimes be marked only through an inhalation or a small cough. This stage could also be marked by other newborn behaviours, including a startle reflex, a sudden opening of the eyes and grasping of the umbilical cord. However, its defining behaviour is the expansion of the lungs and breathing air. The Birth Cry produces a wealth of information about the baby's condition (Branco, Behlau, & Rehder, 2005) and is considered as part of the APGAR score.

The majority of staff interaction with the newborn occurs during this stage. During the period of the Birth Cry, the newborn is dried and placed gently on the semireclined mother's chest. The newborn should be prone, with hands on either side of the body and head turned to the side, to maximize skin contact and warmth. The staff must use gentle hands in order to not hinder the expansion of the newborn's lungs. The umbilical cord is usually long enough to allow the newborn to be placed skin‐to‐skin with the mother without cutting the cord prematurely. After the newborn is settled with the mother, the majority of staff interaction throughout the following hour or so involve monitoring and assessing the newborn, which can occur while the newborn remains skin‐to‐skin with the mother.

In the first minutes after the birth, all but 8 of the 69 newborns in this reanalysis experienced a period of Relaxation. The Relaxation Stage begins after the newborn is settled on the mother's chest. At this point, the newborn stops crying and lays still. This behaviour may be the ‘fear‐induced freezing’, which occurs during a perceived threat or a ‘startle‐induced behavioural arrest’, which occurs prior to a cognitive assessment of a new situation (Roseberry & Kreitzer, 2017). If unexpected, this lack of crying or noise can be disconcerting to the parents or staff. This stage is marked by stillness of the infant—limited movements or actions.

It would be important to re‐examine the context to understand what would be preventing a newborn from entering the Relaxation Stage. In some cases, the lack of noise from the infant can result in vigorous rubbing or massaging of the newborn. This is unnecessary if the newborn is breathing easily and ‘pinking up’, and unneeded interference may prevent the newborn from entering into this calming stage. Staff education may be necessary.

All of the newborns in all four countries experienced the Awakening Stage. The iconic action of the Awakening Stage occurs when the eyes open. However, as with the Birth Cry Stage, which may occur without a cry, this stage can occur without eye opening. Bright lights or labour medications may inhibit newborns from eye opening. Instead, other elements of awakening may be present, for example, small thrusts of the head from side to side or up and down, small movements of the limbs and shoulders and small amounts of mouth activity. The clearest element of this stage is head movements or motion of the shoulder and limbs. Clinical support includes awareness of the room ambiance, such as where lights are shining in relation to the newborn's eyes and protecting the noise level of the room, as well awareness of the medication exposure of the newborn.

All but one of the 68 newborns in four countries experienced the Activity Stage. During the Activity Stage the small, hesitant movements of the Awakening Stage take on more definition and purpose. Arms and legs move purposefully. Often the initiation of the Activity Stage is marked with a sweeping movement of an arm. As the Activity Stage continues, the newborn may look as if they are pushing against the mother (without the motion that would transition the stage to Crawling), protrude the tongue, root against the mother's chest, make soliciting sounds, move the hand to the breast, move the hand to the mouth, or move the hand from the mouth to the breast and back to the mouth. At this point, the newborn has stable open eyes (the eyes stay open for 5 min or more), and the newborn may look at the breast or shift to look at the mother. Activity intensifies in purpose and determination during this stage. It is interesting to note that the mean time of appearance in minutes for the United States newborns is 22 min, while the mean for the Japanese (11 min), Italian (13 min) and Swedish (8 min) newborns are more similar.

The newborn will rest throughout the first hour after birth. This Resting Stage is interspersed with the other stages. The newborn may have periods of activity, then rest, then become active again. Or the Resting Stage may occur between stages, for example, after Activity and before the newborn begins to crawl. These periods of rest may be vital for the newborn's consolidation of learning. The wide range of difference in the period of initial rest may be dependent on research analysis—how much rest constitutes entering a Resting Stage, versus a pause in behaviour? When analysing the American and Japanese newborns, we used the definition of stillness, limited motions, that continued for more than 15 s. Rest was not defined in the Italian study, except to say that the newborn conducts massage of the breast with the fist or open hand during the resting phase. The definition of rest in the Swedish study ‘infant rests, with some activity, such as mouth activity, sucks on hand’. These do not match the definition of the American or Japanese studies.

Clinical support for this stage requires staff education on the importance of rest. It is easy to confuse the concept of resting with the newborn being ‘done’, and this misunderstanding may result in prematurely removing the baby from SSC.

It is often difficult to determine when the Crawling Stage begins. Careful observers may notice that the newborn does not seem to be in the same place any more. ‘Crawling’ could involve slow shifting of the body or full pushes with the legs. The crawling motion may happen when the newborn is in full contact with the mother or involve lifting part of the infant's body off of the mother to thrust in a new direction. It is interesting to note the similarity of initiation of the timing of the Crawling Stage in all four country reports, around half an hour after the birth. Although 9 of the 11 USA newborns, 16 of the 17 Italian newborns and 21 of the 28 Swedish newborns exhibited the Crawling Stage, only 7 of the 13 Japanese newborns crawled to the breast.

Again, it is interesting to note the similarities of initiation timing of the Familiarization Stage in the four different groups, around 40 min after the birth. The Familiarization Stage begins when the newborn arrives at the breast and nipple. This location defines the initiation of the stage. During this stage, the newborn may massage the breast or nipple, move the hand from the mouth to the breast, put the hand or fingers in the mouth, protrude the tongue above, around, near and on the nipple, suck on the nipple, suck on the hand and make soliciting sounds. The newborn may look at the mother or at the breast. During this time, the nipple and breast change shape, as the oxytocin levels increase, and the breast prepares for suckling. The behaviour of the newborn's tongue changes during this time as well. It may take 20 min or more for the newborn to progress through this stage. It is important not to rush or ‘help’ the newborn during this time. Research shows that ‘helping’ a newborn to breastfeed during this first hour may be related to later breastfeeding difficulties (Svensson et al., 2013).

The newborn will self‐attach and suckle around 50 min after the birth. During this first experience of suckling, the ‘usual rules’ of breastfeeding—nose to nipple, gape, asymmetric latch—do not apply. The newborn's latch will be appropriate for this early learning experience of suckling colostrum. The newborn may begin to make soliciting sounds, lick, suckle and then adjust the mouth‐to‐nipple contact several times to optimize the suckling position. The newborns in the United States, Japan and Italy data are unmedicated and self‐attach at a median of 52, 52 and 45 min. It is interesting to note that the Swedish newborns self‐attach around 62 min, which may be associated with the labour medication exposure of those infants (Brimdyr et al., 2015; Nissen et al., 1997; Ransjö‐Arvidson et al., 2001).

Newly born infants fall asleep 1.5 to 2 h after birth (Emde, Swedberg, & Suzuki, 1975). Both the United States study and the Japanese study to only record the first hour after birth means that many of the newborns in those studies were not recorded reaching the Sleeping Stage. Not surprisingly, only one of the 11 American newborns fell asleep before the first hour ended, at 59 min after birth, and none of the Japanese newborns who initiated suckling fell asleep in that time. Indeed, the Italian newborns fell asleep around 94 min after birth, and the Swedish newborns around 70 min after birth. Clinical consideration of the Sleep Stage could influence the initiation of the safe transitioning the dyad from the labour and delivery ward to the post‐partum ward.

## 4.1 Limitations

This study is limited to the four studies which reported on the nine stages in relation to healthy newborn babies, born vaginally. Although the ward routines were not standardized, the reported differences in newborn behaviour was very small. Additionally, the United States study and the Japanese study only recorded the first hour after birth, which means that behaviours that occurred after the first 60 min were not recorded or included. In addition, the Swedish study included 10 mothers who had been exposed to meperidine (Pethidine hudrochloride, ACO Pharmacia).

A limitation of this study is that the majority of Japanese videos followed their interpretation of the older World Health Organization definition of Step 4, which invited staff to ‘Help mothers initiate breastfeeding within a half hour of birth’ (World Health Organization & UNICEF, 1989), and therefore, innate newborn behaviour was not observed after 30 min, since newborns had been ‘helped’ to initiate breastfeeding.

Both the Japanese and American data were scored on the chart (Table 2); it is assumed that the others used similar specifications, although that is not known. However, Table 2 is based on the work of the Swedish research cited here. We also postulate that the Italian research was based on this Swedish work. Therefore, all methods of analysis are based on initial reports of the nine stages in Widström's seminal work (Widström et al., 2011), which has recently been enhanced and strengthened (Widström et al., 2019). Despite this unknown, the similarity of timing is noteworthy.

## 5 CONCLUSION

The comparison of four data sets from around the world—Japan, Sweden, Italy and the United States—consolidates and affirms the existence and timing of Widström's original nine stages. The evidence supports making a safe space and protected time for this newborn behaviour. The evidence supporting SSC during the first hour is so compelling that the 2018 revision of the WHO/UNICEF *Ten Steps to Successful Breastfeeding* which form the basis of the Baby‐Friendly Hospital Initiative state ‘Facilitate immediate and uninterrupted skin‐to‐skin contact and support mothers to initiate breastfeeding as soon as possible after birth’ (World Health Organization, 2018) with the understanding that the newborn will self‐attach. This new wording is more reflective of the process and clinical expectations, as well as highlighting the newborn's ability to reach the breast, self‐attach and breastfeed.

Clinical practices should protect these first hours after birth, until the newborn goes through the nine stages, self‐attaches, suckles and falls asleep. The new Sign of the Stages table can assist in further research concerning this important time. Although the nine stages represent observable behaviour that should be seen in all babies, further research is needed to verify this timing beyond full‐term, healthy and vaginal birth newborns.

## CONFLICTS OF INTEREST

The authors declare that they have no conflicts of interest.

## CONTRIBUTIONS

All authors contributed to the final version of the paper. The paper was primarily written by AW, KB, KC, EN and KS. KB and YT analysed the Japanese data. KB and KC analysed the US data.
